# Noninvasive platelet membrane‐coated Fe_3_O_4_ nanoparticles identify vulnerable atherosclerotic plaques

**DOI:** 10.1002/SMMD.20240006

**Published:** 2024-06-04

**Authors:** Yuyu Li, Yujie Wang, Zequn Xia, Yangjing Xie, Daozheng Ke, Bing Song, Dan Mu, Ronghui Yu, Jun Xie

**Affiliations:** ^1^ Department of Cardiology National Cardiovascular Disease Regional Center for Anhui the First Affiliated Hospital of Anhui Medical University Hefei China; ^2^ Beijing Institute of Heart, Lung, and Blood Vessel Diseases Beijing Anzhen Hospital Affiliated to Capital Medical University Beijing China; ^3^ Department of Radiology Nanjing Drum Tower Hospital Affiliated Hospital of Medical School Nanjing University Nanjing China; ^4^ Department of Cardiology Nanjing Drum Tower Hospital Affiliated Hospital of Medical School Nanjing University Nanjing China; ^5^ Department of Medicine Cardiovascular Center Medical College of Wisconsin Milwaukee Wisconsin USA; ^6^ Department of Radiology Nanjing Drum Tower Hospital Clinical College of Jiangsu University Nanjing China

**Keywords:** Fe_3_O_4_nanoparticle, MRI, platelet membrane, vulnerable atherosclerosis plaques

## Abstract

Vulnerable atherosclerotic plaques serve as the primary pathological basis for fatal cardiovascular and cerebrovascular diseases. The precise identification and treatment of these vulnerable plaques hold paramount clinical importance in mitigating the incidence of myocardial infarction and stroke. Nevertheless, the identification of vulnerable plaques within the diffuse atherosclerotic plaques dispersed throughout the systemic circulation continues to pose a substantial challenge in clinical practice. Double emulsion solvent evaporation method, specifically the water‐in‐oil‐in‐water (W/O/W) technique, was employed to fabricate Fe_3_O_4_‐based poly (lactic‐co‐glycolic acid) (PLGA) nanoparticles (Fe_3_O_4_@PLGA). Platelet membranes (PM) were extracted through hypotonic lysis, followed by ultrasound‐assisted encapsulation onto the surface of Fe_3_O_4_@PLGA, resulting in the formation of PM‐coated Fe_3_O_4_ nanoparticles (PM/Fe_3_O_4_@PLGA). Characterization of PM/Fe_3_O_4_@PLGA involved the use of dynamic light scattering, transmission electron microscopy, western blotting, and magnetic resonance imaging (MRI). A model of atherosclerotic vulnerable plaques was constructed by carotid artery coarctation and a high‐fat diet fed to ApoE^−/−^ (Apolipoprotein E knockout) mice. Immunofluorescence and MRI techniques were employed to verify the functionality of PM/Fe_3_O_4_@PLGA. In this study, we initially synthesized Fe_3_O_4_@PLGA as the core material. Subsequently, a platelet membrane was employed as a coating for the Fe_3_O_4_@PLGA, aiming to enable the detection of vulnerable atherosclerotic plaques through MRI. In vitro, PM/Fe_3_O_4_@PLGA not only exhibited excellent biosafety but also showed targeted collagen characteristics and MR imaging performance. In vivo, the adhesion of PM/Fe_3_O_4_@PLGA to atherosclerotic lesions was confirmed in a mouse model of vulnerable atherosclerotic plaques. Simultaneously, PM/Fe_3_O_4_@PLGA as a novel contrast agent for MRI has shown effective identification of vulnerable atherosclerotic plaques. In terms of safety profile in vivo, PM/Fe_3_O_4_@PLGA has not demonstrated significant organ toxicity or inflammatory response in the bloodstream. In this study, we successfully developed a platelet‐membrane‐coated nanoparticle system for the targeted delivery of Fe_3_O_4_@PLGA to vulnerable atherosclerotic plaques. This innovative system allows for the visualization of vulnerable plaques using MRI, thereby demonstrating its potential for enhancing the clinical diagnosis of vulnerable atherosclerotic plaques.

## Graphical abstract

Vulnerable plaques associated with atherosclerosis are the primary pathological foundation of fatal cardiovascular and cerebrovascular ailments, and the rupture of vulnerable plaques can result in sudden death. Vulnerable plaque‐targeted MR molecular imaging to facilitate identification remains a major challenge because of the systemic occurrence of atheroma. Here, we constructed Fe_3_O_4_‐based poly(lactic‐co‐glycolic acid) (PLGA) nanoparticles. A platelet membrane was used to coat Fe_3_O_4_ nanoparticles (PM/Fe_3_O_4_ NPs) for the detection of vulnerable atherosclerotic plaques. In vivo, the adhesion of platelet membrane‐coated nanoparticles to atherosclerotic lesions was confirmed in a mouse model of vulnerable atherosclerotic plaques. Intravenous injections of the hybrid system successfully identified vulnerable atherosclerotic plaques. This strategy provides a potential diagnostic system for the targeted delivery of Fe_3_O_4_ to vulnerable atherosclerotic plaques for the diagnosis of atherosclerosis in CVD patients.

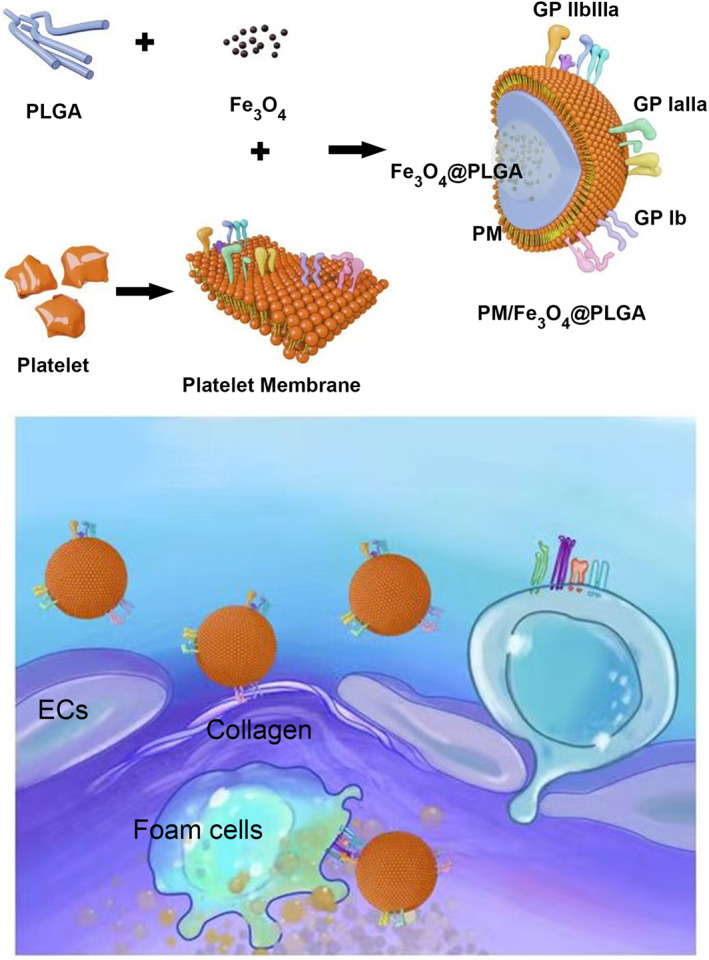

**Key points**

- In this work, PM/Fe_3_O_4_@PLGA were prepared with a W/O/W method, which allowed water‐soluble MRI contrast agents to be efficiently loaded and transported in vivo.
- PM/Fe_3_O_4_@PLGA exhibited the properties of good targeting, biocompatibility, and safety.
- The biomimetic biofilm‐coated MRI contrast agents were utilized for diagnosing atherosclerotic vulnerable plaques for the first time.
- PM/Fe3O4@PLGA could effectively identify atherosclerotic vulnerable plaques, which showed the potential clinical application value.

## 1 INTRODUCTION

Ischemic cardiovascular and cerebrovascular diseases, primarily attributed to the rupture of vulnerable atherosclerotic plaques, remain the leading cause of mortality worldwide.^1^, ^2^, ^3^, ^4^ Vulnerable plaques are atheromatous plaques that are prone to becoming culprit plaques and characterized by the accumulation of lipids, inflammatory cell aggregation, and extracellular matrix development.^1^, ^5^, ^6^, ^7^

In clinical practice, precise detection of vulnerable plaques is crucial to prevent artery occlusion and provide sufficient evidence for intra‐arterial treatments, such as balloon angioplasty and stenting. Several methods are currently employed for detecting atherosclerotic plaques. Ultrasound and optical coherence tomography have proven effective in identifying intima‐media protrusions, plaque area, or volume. Computed tomography with an iodine contrast agent can detect and classify plaques into three categories based on their composition: calcified, noncalcified, or a mixture of both.^8^, ^9^, ^10^ However, the identification of vulnerable atherosclerotic plaques remains a significant challenge. Therefore, there is an urgent need for the development of novel methods capable of accurately identifying vulnerable atherosclerotic plaques.

Biomimetic nanomaterials have recently gained significant attention as contrast agents in the field of medical imaging.^11^, ^12^, ^13^, ^14^, ^15^, ^16^ In the context of vulnerable atherosclerotic plaques, platelets adhere to damaged endothelium through interactions involving von Willebrand factor (vWF)‐platelet glycoprotein (GP) Ibα, collagen‐platelet GPIa‐IIa, and GP VI.^17^ Given the pivotal spatiotemporal role of platelets in atherosclerosis, the utilization of platelet‐inspired agents holds great promise for precise targeting of vulnerable plaques, thereby enhancing imaging efficacy.^18^, ^19^, ^20^ Magnetic resonance angiography, employing gadolinium‐diethylenetriaminepentaacetic acid as a representative agent,^21^ enables accurate assessment of large arteries and veins with high spatial resolution. Notably, Fe_3_O_4_ has recently emerged as an alternative contrast agent in Magnetic resonance angiography, demonstrating excellent imaging characteristics and a superior safety profile compared to gadolinium‐based agents.^22^, ^23^, ^24^, ^25^ In this study, we have developed a novel contrast agent by coating biomimetic Fe_3_O_4_‐based poly (lactic‐co‐glycolic acid) (PLGA) nanoparticles (Fe_3_O_4_@PLGA) with a platelet membrane, enabling accurate detection of vulnerable atherosclerotic plaques using magnetic resonance imaging (MRI) T2‐weighted images.

In this study, we achieved successful synthesis of PM‐coated Fe_3_O_4_ nanoparticles (PM/Fe_3_O_4_@PLGA) with a pronounced T2 effect. Subsequent in vitro experiments were conducted to evaluate the physiological functionalities of the PM/Fe_3_O_4_@PLGA. Additionally, the PM/Fe_3_O_4_@PLGA exhibited successful detection of vulnerable plaques in an experimental mouse model utilizing MRI. The development of this novel biomimetic contrast agent holds promising clinical applications in the diagnosis of vulnerable atherosclerotic plaques.

## 2 RESULTS AND DISCUSSION

### 2.1 Platelets were the best candidate for adherent vulnerable plaques

**FIGURE 1:**
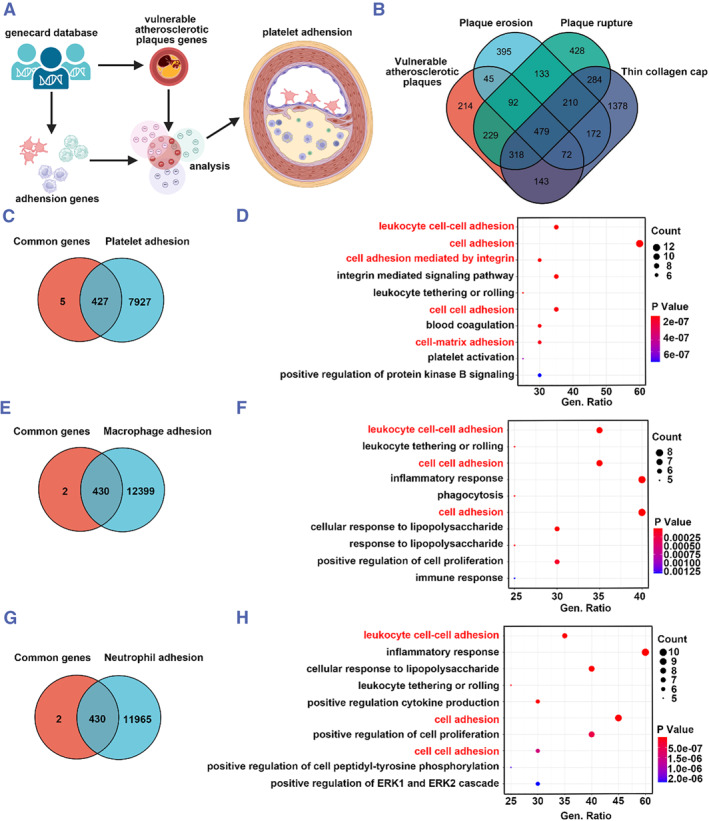
Platelets were the best candidates for adherent vulnerable plaques. (A) Schematic diagram of comprehensive analysis of best candidate adhesion to for vulnerable atherosclerotic plaques. (B) Venn diagrams of vulnerable atherosclerotic plaques, plaque erosion, plaque rupture, and thin collagen cap related gene from GENCARDS. Venn diagrams of vulnerable atherosclerotic plaque common genes and Platelet Adhesion (C), Macrophage adhesion (E), and Neutrophil adhesion (G), respectively. GO pathway enrichment analysis of the overlap genes from Platelet adhesion (D), Macrophage adhesion (F), and Neutrophil adhesion (H).

In recent years, nano‐drug delivery systems based on natural biofilms (macrophage, neutrophil and platelet membranes) have been progressively applied in the treatment of various cardiovascular diseases, especially atherosclerosis. However, it is still uncertain which biofilm is the best candidate for nano‐diagnostics and therapeutics for atherosclerotic vulnerable plaques. The biological interactions and adhesion between atherosclerotic vulnerable plaque and biofilm are important factors in selecting the optimal biofilm. Therefore, a comprehensive analysis was conducted to explore the potential candidate targeting atherosclerotic vulnerable plaques for nano‐diagnostics (Figure 1A). Based on atherosclerotic vulnerable plaque characteristics, a total of 1592 vulnerable atherosclerotic plaques‐related genes, 1598 genes related to plaque erosion, 2173 genes related to plaque rupture, and 3056 genes related to thin collagen cap were extracted from GENECARDS (https://www.genecards.org/). These datasets were imported into Hiplot (https://hiplot.com.cn/home/index.html). 479 common genes were identified and displayed in Venn diagrams (Figure 1B). In addition, a total of 8354, 12,829 and 12,395 genes were extracted based on platelet adhesion, macrophage adhesion and neutrophil adhesion. In order to explore the optimal biofilm for adhesion to vulnerable plaques, the adhesion genes of these different kinds of cells were compared with the common genes that characterize vulnerable plaques, from which we found that they have 427, 430, and 430 genes in common, respectively. 5.1%, 3.35% and 3.47% of their respective total gene counts, respectively (Figure 1C,E,G). Further, we performed GO pathway enrichment analysis on these overlapping genes using the cluster Profiler R package. Figure 1D,F,H show the 10 most relevant pathways in each group. Of the 10 pathways of platelet adhesion, a total of 5 are all associated with adhesion, namely leukocyte cell‐cell adhesion, cell adhesion, cell adhesion mediated by integrin, cell‐cell adhesion and cell‐matrix adhesion. In addition, the top three pathways were also associated with adhesion. Of the overlapping genes for macrophage adhesion and neutrophil adhesion, only three were associated with the adhesion pathway. These findings showed that platelets were the best candidates for adherent vulnerable plaques.

### 2.2 Syntheses and characteristics of the platelet‐coated Fe_3_O_4_ nanoparticles

**FIGURE 2:**
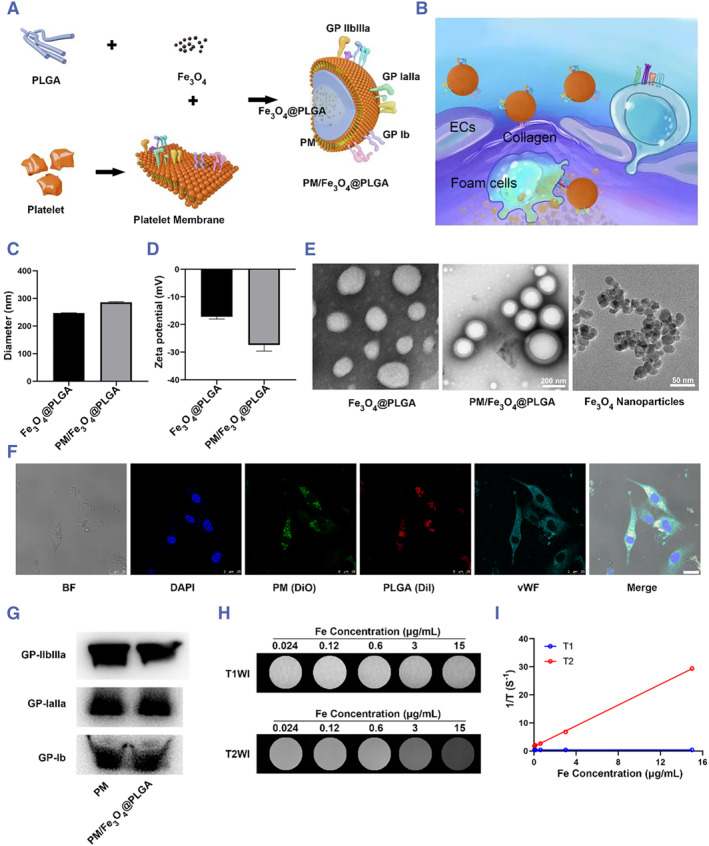
Preparation and characterization of PM/Fe_3_O_4_@PLGA. (A) Schematic illustration of the preparation of PM/Fe_3_O_4_@PLGA. The membrane of platelet was extracted using a hypotonic lysis method. PM and Fe_3_O_4_@PLGA were mixed and extruded to formulate PM/Fe_3_O_4_@PLGA. (B) Schematic diagram of vulnerable atherosclerotic plaque targeting performance of PM/Fe_3_O_4_@PLGA in the blood vessels. (C) Particle size of Fe_3_O_4_@PLGA and PM/Fe_3_O_4_@PLGA measured by DLS. (D) Zeta potential distribution of Fe_3_O_4_@PLGA and PM/Fe_3_O_4_@PLGA. (E) TEM images of Fe_3_O_4_@PLGA and PM/Fe_3_O_4_@PLGA. (Scale bar = 100 nm). (F) CLSM images of ECs after incubation with PM/Fe_3_O_4_@PLGA for 4 h. The nuclei of ECs were stained with DAPI. The PM “shell” was labelled with DiO and the PLGA core was labelled with DiI. The merged image is the overlay of the four individual images (Scale bar = 25 μm). (G) Representative protein bands of PM and PM/Fe_3_O_4_@PLGA measured by western blot. (H) MRI images of different concentration aqueous solutions of PM/Fe_3_O_4_@PLGA. (I) T1 and T2 relaxation times of PM/Fe_3_O_4_@PLGA.

As mentioned above, the platelet membrane (PM) was selected as an optimal coating membrane candidate due to its inherent affinity for vulnerable plaques and its natural presence in atherosclerotic lesions (Figure 2B).^26^, ^27^ To prepare PM/Fe_3_O_4_@PLGA, Fe_3_O_4_‐loaded PLGA nanoparticles (Fe_3_O_4_@PLGA) were initially synthesized, followed by coating the Fe_3_O_4_@PLGA with the PM using an extrusion method (Figure 2A). Dynamic light scattering (DLS) analysis revealed that the diameter of the nanoparticles increased from approximately 244.13 to 280.33 nm after the PM coating step, corresponding to the thickness of the bilayer membrane (Figure 2C). The zeta potentials of the PM and PM/Fe_3_O_4_@PLGA were measured as −17.2 ± 0.67 and −27.36 ± 1.84 mV, respectively, which suggests prolonged blood circulation compared to nanoparticles with neutral or positive surface charges (Figure 2D). Transmission electron microscopy (TEM) further confirmed the “core‐shell” structure of the PM/Fe_3_O_4_@PLGA (Figure 2E).

To assess the coating of nanoparticles with the platelet membrane, 1,1′‐Dioctadecyl‐3,3,3′,3′‐tetramethylindocarbocyanine perchlorate (DiI) fluorophore was incorporated into the PLGA “core” instead of Fe_3_O_4_, while the platelet membrane was labeled with 3,3′‐dioctadecyloxacarbocyanine perchlorate (DiO). Confocal laser scanning microscopy (CLSM) analysis demonstrated the colocalization of DiI‐labeled PLGA “core” and DiO‐labeled platelet membrane, confirming the successful coating of the platelet membrane on the DiI nanoparticles following the extrusion process (Figure S2, Supporting Information). At the site of vulnerable atherosclerotic plaques, ECs were significantly activated, and their adhesion ability was greatly enhanced, enabling them to effectively first contact and adhere PM/DiI@PLGA at the site of vulnerable atherosclerotic plaques. To further investigate the stability of the platelet membrane‐coated DiI nanoparticles (DiI@PLGAPM/DiI@PLGA) within target cells after endocytosis, DiO‐labeled DiI@PLGAPM/DiI@PLGA were incubated with human umbilical vein endothelial cells (ECs) for 4 h. The majority of DiI and DiO signals were observed to colocalize within the cells (Figure 2F), indicating the robust stability of the PM/Fe_3_O_4_@PLGA following internalization by the cells. Collectively, these findings provide evidence for the successful coating of PLGA cores with the extracted platelet membrane.

Next, we sought to test the key physiological functions of PM/Fe_3_O_4_@PLGA inherited from the platelet membrane. Western blot analysis was performed to detect specific protein signals of GP‐IIbIIIa, GP‐IaIIa, and GP‐Ib, which are crucial for platelet adhesion to injured tissue. The presence of these specific protein signals confirmed the preservation of platelet function on the PM/Fe_3_O_4_@PLGA following membrane coating (Figure 2G). In addition, the protein composition of Fe_3_O_4_@PLGA, platelet membrane and PM/Fe_3_O_4_@PLGA were also shown by Ponceau dyeing (Figure S1). Furthermore, to assess the feasibility of Fe_3_O_4_ as an MRI contrast agent, T1 and T2 relaxation values of Fe_3_O_4_ at various Fe_3_O_4_ concentrations were measured. T1 and T2 relaxation values showed that the ratio of r2 to r1 was relatively high for the PM/Fe_3_O_4_@PLGA (PM/Fe_3_O_4_@PLGA: r2/r1 = 684.81, r2/r1 > 10), indicating a strong T2 effect (Figures 2H,I).

### 2.3 Imitating platelet adhesive characteristics in vitro

**FIGURE 3:**
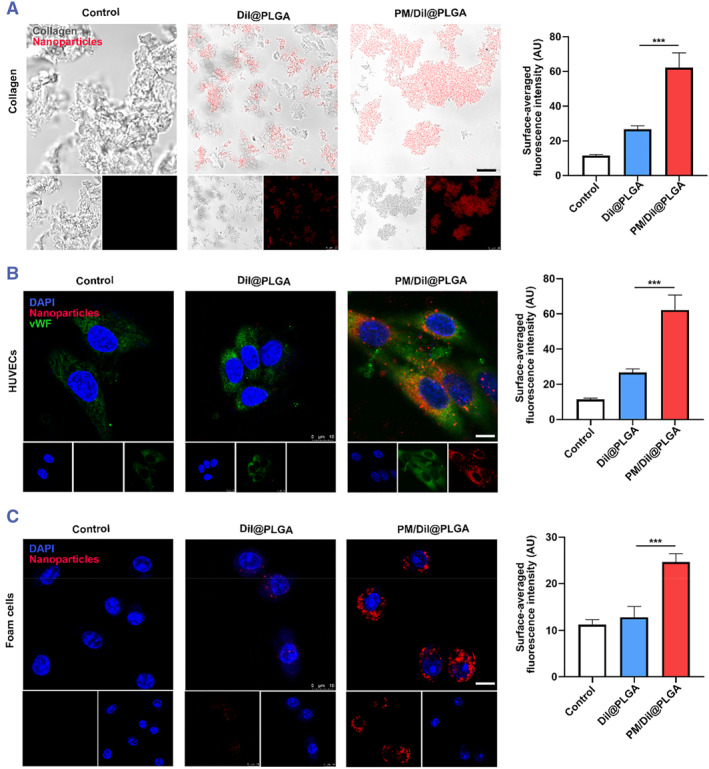
Targeted delivery performance of PM NPs. (A) The ability of collagen adheres with DiI@PLGA and PM/DiI@PLGA after 4 h incubation with in vitro collagen (gray is collagen, red is encapsulated DiI fluorescent molecules, scale bar = 10 μm). (B) Cell uptake of Dil NPs and PM/Dil NPs after incubation with foam cells for 4 h. The nuclei of foam cells were stained with DAPI (scale bar = 10 μm). (C) Cell uptake of Dil NPs, MM/Dil NPs and MMM/Dil NPs after incubation with activated HUVECs (stimulated with TNF‐α) for 4 h. The nuclei of HUVECs were stained with DAPI (scale bar = 10 μm). (Mean ± SD, *n* = 3 independent experiments). ***p < 0.001, one‐way ANOVA, Tukey's multiple comparison test.

The targeting of platelets to disease sites involves the interaction between multiple substrates and adhesive factors present on platelet membranes. In this study, we assessed the adhesive properties of the DiI@PLGAPM/DiI@PLGA by examining their ability to bind to collagen and von Willebrand factor (vWF), as well as their uptake by foam cells in vitro. To evaluate the binding ability of the DiI@PLGAPM/DiI@PLGA to collagen, a flow chamber study was conducted at a shear rate of 500/s. The results demonstrated that the PM/DiI_4_ NPs exhibited significant adhesion to collagen, whereas the DiI@PLGA showed minimal binding to collagen (Figure 3A). To evaluate the binding efficacy of PM/NPs to von Willebrand factor (vWF), human umbilical vein endothelial cells (HUVECs) were stimulated with Recombinant human Tumor Necrosis Factor (TNF‐*α*) to induce vWF expression. Subsequently, the cells were treated with DiI@PLGAPM/DiI@PLGA and DiI@PLGA for 4 h. The PM/DiI NP group exhibited strong red fluorescence signals on the cell surface, whereas only green fluorescence corresponding to vWF was observed in the non‐targeted DiI NP group (Figure 3B).

Platelets play a crucial role in guiding inflammatory cells to atherosclerotic vulnerable plaques through the formation of platelet‐mononuclear complexes, which can be taken up by foam cells. Therefore, we examined the cellular uptake of DiI@PLGAPM/DiI@PLGA by foam cells in vitro. RAW 264.7 cells were treated with Ox‐LDL to stimulate foam cell formation. Fluorescence imaging demonstrated a significantly higher accumulation of DiI@PLGAPM/DiI@PLGA within foam cells compared to DiI@PLGA (Figure 3C). These results suggest that DiI@PLGAPM/DiI@PLGA exhibit adhesive properties similar to platelets, thereby facilitating their targeted binding to vWF and uptake by foam cells.

### 2.4 Detection of vulnerable atherosclerotic plaques by PM/Fe_3_O_4_@PLGA in vivo

**FIGURE 4:**
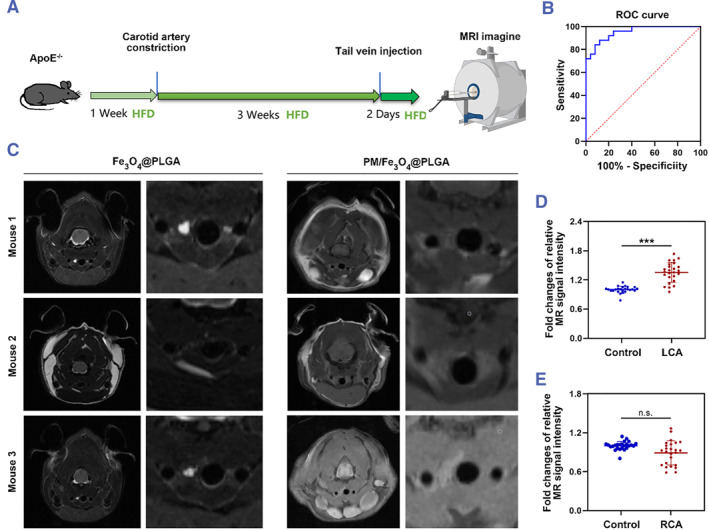
MRI of the vulnerable atherosclerotic plaque in vivo. (A) Experiment setup in this study. (B) Receiver Operating Characteristic (ROC) curve showing the performance of different plaques (stable plaque, venerable plaque) in terms of true positive rate and false positive rate. (C) MRI images were taken 24 h after injection. Red arrows point to atherosclerosis vulnerable plaque (*n* = 25). (D, E) Quantitative analysis of relative MR signal intensity in atherosclerotic mice treated with PM/Fe_3_O_4_@PLGA was conducted through a RadiAnt DICOM Viewer (mean ± SD, *n* = 25 independent experiments). ***p < 0.001, LCA, left carotid artery; n.s., not significant; RCA, right carotid artery; *t* test.

To investigate the capability of PM/Fe_3_O_4_@PLGA in detecting vulnerable atherosclerotic plaques, a mouse model of atherosclerosis characterized by plaque instability/rupture was established (Figure 4A). Coarctation surgery was performed on the left carotid artery (LCA) of mice, while the right carotid arteries (RCA) remained untreated. The induction of vulnerable plaques in the LCA was facilitated by a high‐fat diet, whereas stable atherosclerotic plaques were observed in the RCA. In this study, ApoE^−/−^ mice with vulnerable atherosclerotic plaques were administered PM/Fe_3_O_4_@PLGA or Fe_3_O_4_@PLGA (0.72 mg/kg Fe) via the tail vein. MRI was conducted over a 24‐h period to visualize the vulnerable atherosclerotic plaques in the mouse carotid artery (Figure 4A). Transverse plane images revealed a distinct MR signal in the LCA of the atherosclerotic mouse that received PM/Fe_3_O_4_@PLGA 24 h post‐injection, while no significant changes in the MR signal were observed in atherosclerotic mice treated with Fe_3_O_4_@PLGA. Additionally, no signal was detected in the RCA of either treatment group (Figure 4C–E). These findings indicate the specific targeting ability of PM/Fe_3_O_4_@PLGA towards vulnerable atherosclerotic plaques in vivo.

Furthermore, to assess the diagnostic potential of PM/Fe_3_O_4_@PLGA in identifying vulnerable plaques, 25 ApoE^−/−^ mice with vulnerable atherosclerotic plaques underwent MRI, yielding an area under the curve (AUC) of 0.954 ± 0.026. Receiver Operating Characteristic (ROC) analyses revealed an optimal cutoff of 8493.5, resulting in 88% sensitivity and 88% specificity (Figure 4B). These results demonstrate the excellent diagnostic efficacy of PM/Fe_3_O_4_@PLGA for vulnerable atherosclerotic plaques in vivo.

### 2.5 Targeting effect of PM/Fe_3_O_4_@PLGA to vulnerable plaque

**FIGURE 5:**
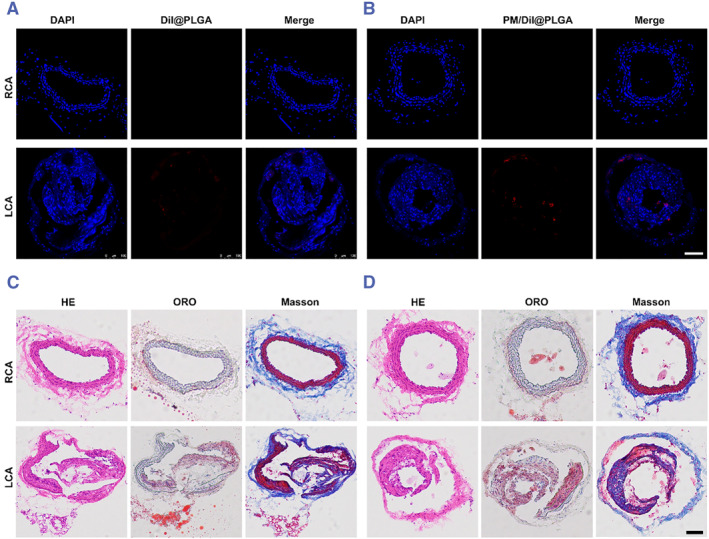
PM NPs mediated delivery to the vulnerable atherosclerotic plaque mice and H&E, Oil Red O and Masson‐stained vulnerable atherosclerotic plaque. (A), (B) CLSM images of different Dil labelled nanoparticles in atherosclerotic plaque of LCA and RCA in the vulnerable atherosclerotic plaque mice at 24 h post intravenous injection (scale bar = 100 μm). The cell nuclei were stained with DAPI and different nanoparticles were labelled with DiI. (C), (D) H&E, Oil Red O and Masson‐stained vulnerable atherosclerotic plaque (scale bar = 100 μm). LCA, left carotid artery; RCA, right carotid artery.

Subsequently, we assessed the in vivo targeting capability of PM nanoparticles towards vulnerable plaques in a mouse model by histological analysis. For CLSM analysis, DiI@PLGAPM/DiI@PLGA was employed. An equal number of DiI@PLGA and DiI@PLGAPM/DiI@PLGA were intravenously injected into mice with vulnerable atherosclerotic plaques. Frozen sections of the plaques in the LCA and RCA were examined 24 h post‐injection. Minimal fluorescence signal was observed in the vulnerable plaque following injection of DiI@PLGA in the LCA, whereas sections from PM/DiI‐NP‐treated mice exhibited abundant fluorescence signal, indicating enhanced targeting of the vulnerable plaque by PM NPs (Figure 5A,B). In addition, H&E, Oil Red O and Masson‐stained RCA and LCA are also shown in Figure 5C,D.

Here, DiI@PLGAPM/DiI@PLGA was shown to effectively accumulate in these regions through CLSM analysis. Meanwhile, in vivo MRI of the mice confirmed the ability of the PM/Fe_3_O_4_@PLGA to recognize atherosclerotic vulnerable plaques above. These findings could lead to the identification of vulnerable atherosclerotic plaques for clinical diagnosis.

### 2.6 Biosafety assignment

To assess the potential biological impact of PM nanoparticles, we exposed ECs, smooth muscle cells (SMCs) and macrophages to the PM nanoparticles, as well as to PLGA nanoparticles. The cytotoxicity of these materials was evaluated using the Cell Counting Kit‐8 (CCK‐8) assay. As depicted in Figure S3, no significant differences were observed between the various treatment groups and the control group that was not exposed to any nanoparticles. These findings indicate that our drug delivery system is well tolerated by cells and exhibits favorable cytocompatibility.

Moreover, blood compatibility tests demonstrated that the different PLGA nanoparticles and PM NPs did not induce significant hemolysis (Figure S4). These results further support the good biocompatibility and biosafety of the prepared PM NPs.

In vivo, histological examination of various organ sections using H&E staining revealed no notable differences between the control group and the different treatment groups of mice with vulnerable atherosclerotic plaques (Figure S5). Furthermore, analysis of cytokine levels (TNF‐α, Interleukin‐1β (IL‐1β), Interleukin‐6 (IL‐6), and C‐C motif chemokine ligand 2 (CCL2)) in mouse serum did not display any significant differences between the treatment groups and the control group (Figure S6). These findings suggest that PM/Fe_3_O_4_@PLGA and Fe_3_O_4_@PLGA exhibit high biocompatibility after long‐term treatment in mice with vulnerable atherosclerotic plaques.

## 3 CONCLUSIONS

In the present study, we have successfully developed a nanoparticle system coated with platelet membranes for the targeted delivery of Fe_3_O_4_ nanoparticles to vulnerable atherosclerotic plaques, enabling the visualization of these plaques through MRI. Western blot indicated that the key proteins on the platelet membrane surface that mediate adhesion to collagen are not lost during the membrane extraction and re‐encapsulation process in vitro. Furthermore, functional experiments provided further evidence that the platelet membrane encapsulated on the NPs retained its ability to adhere to collagen and induce macrophage cellular uptake. This novel formulation was also found to be a safe and effective in vivo MRI contrast agent.

In this study, the results demonstrated that PM/Fe_3_O_4_ nanoparticles exhibited excellent biocompatibility and possessed the ability to target atherosclerosis‐prone plaque lesions. A major advantage of our PM NP delivery system is the advanced targeting ability in response to biological signals in the process of vulnerable atherosclerosis compared to the traditional nanoparticle delivery system. This innovative approach expands the application of biomimetic platelet membrane‐modified nanoparticles and holds great potential for clinical diagnosis of vulnerable atherosclerotic plaques.

## 4 EXPERIMENTAL SECTION

*Materials and Cells*: The anti‐human vWF antibody was bought from Abclonal (Changsha, China), and the anti‐mouse GP‐Iba antibody was obtained from ProMab (USA). PLGA (75:25; MW = 20,000) and an aqueous solution of Fe_3_O_4_ particles were purchased from Dalian Meilun Biotechnology (Dalian, China). Polyvinyl alcohol (PVA) (MW = 30,000−70,000) was purchased from Aladdin Bio‐Chem Technology (Shanghai) Co., Ltd. (Shanghai, China). DiI and DiO were purchased from Beyotime (Beijing, China). TNF‐α was purchased from Novoprotein Scientific. Inc. (Canada). Servicebio (Wu Han, China) provided hematoxylin and eosin, Masson's trichrome, and Oil Red O (ORO). Ponceau S, a Cell Counting Kit 8 (CCK‐8) and a Bicinchoninic Acid Assay (BCA) protein assay kit were purchased from Biosharp (China). Mouse TNF‐*α* and IL‐6 ELISA kit was purchased from LunchangshuoBiotech (Xiamen, China). Mouse CCL2 ELISA kit was purchased from Fankew (Shanghai, China) and mouse IL‐1*β* ELISA kit was purchased from Jingmei Biological Technology Co.,Ltd (Jiangsu, China).

The murine RAW 264.7 cell line, the murine SMCs and HUVECs were purchased from Beinart Biotech (Beijing, China).

*Construction of Pathways*. Vulnerable atherosclerotic plaques‐related genes and cell adhesion genes were retrieved from GeneCards (https://www.genecards.org/). Vene diagrams showing common genes were designed in Hiplot (https://hiplot.com.cn/home/index.html). The common gene profiles were analyzed using the bioconductor packet. R were used for GO enrichment analysis.

*Isolation of the PM*. The platelet membrane was generated using a repeated freeze‒thaw process as previously described.^28^ The platelets were subjected to hypotonic lysis to extract a biologically active membrane. The hypotonic lysate solution was prepared as previously described.^29^ The protein concentration was measured using the BCA protein assay kit.

*Preparation of Fe*_*3*_ *O*_*4*_ *@PLGA:* We employed the nanoprecipitation method to fabricate Fe3O4 nanoparticles, following a previously established protocol. In summary, we dissolved 100 mg of PLGA in dichloromethane and then added 0.2 mL of an aqueous solution containing Fe3O4 particles (1 mg/mL). The mixture underwent 60 s of acoustic vibration using an ultrasonic oscillation instrument, resulting in the formation of a brown emulsion. The emulsion was homogenized at 15,000 rpm for 5 min in a 10 mL 3% PVA solution using a high‐speed homogenization dispersion machine. The nanoparticle surface solidified as dichloromethane was volatilized over 2 h at room temperature, followed by the addition of 20 mL of a 2% isopropanol solution. Similarly, DiI‐labeled NPs were prepared in the same manner but with the inclusion of 0.1 wt% DiI.

*Preparation of PM/Fe*_*3*_ *O*_*4*_ *@PLGA:* To prepare platelet membrane vesicles, the extracted membrane was sonicated for 20 min. After mixing the platelet vesicles with Fe_3_O_4_@PLGA, sonicating, and extruding through 200 nm‐pore membrane filters, the pellets were reconstituted.

The hydrodynamic size of Fe3O4 nanoparticles as well as the zeta potential of PM/Fe3O4 nanoparticles were measured using DLS (Malvern Panalytical, England). Morphologies were determined by TEM (FEI Tecnai F20, America).

*Flow chamber experiment for collagen binding analysis*: A microscope slide was coated with 20 μL of collagen solution type IV (2.0 mg/mL in 0.25% acetic acid) at 4°C. Flow chambers were then filled with DiI‐labeled PM/Fe3O4 or Fe_3_O_4_@PLGA (5 × 10^6^/mL) suspended in PBS for 3 min, followed by a 5‐min washing period. CLSM (Leica, Germany) was used to measure nanoparticle adhesion to collagen.

*Cell uptake* in vitro: RAW 264.7 cells were treated with Ox‐LDL to stimulate foam cell formation. The cells were cultured overnight. DiI@PLGA and DiI@PLGAPM/DiI@PLGA (150 μg per well) were added and cultured for 4 h. Subsequently, the cell nuclei were stained with DAPI and observed using CLSM.

Following exposure to 50 ng/mL TNF‐α (Gibco) for 24 h, the HUVECs were treated with either 100 μg of DiI@PLGA or PM/Dil NPs, depending on their activation status. After 2 h of incubation, the uptake efficiencies of the DiI@PLGAPM/DiI@PLGA were measured using CLSM. The cell nuclei were stained with DAPI and analyzed using CLSM.

*Cytotoxicity test:* The types of cells were plated in a 96‐well plate at a density of 1 × 10^4^ cells/well and allowed to incubate for about 6 h. Subsequently, the cells were treated with PM/NPs for 24 h, and cell viability was assessed using CCK‐8 assays.

*Blood compatibility tests:* One milliliter of sodium chloride solution was obtained by diluting with 1.25 mL of rat blood. Subsequently, 100 μL of diluted whole blood was added to a 5 mL solution of Fe_3_O_4_@PLGA or PM/Fe_3_O_4_@PLGA and incubated for 1 h at 37°C, followed by centrifugation at 3000 rpm for 5 min. The release of hemoglobin from lysed red blood cells was measured from the supernatant at 540 nm.

*Mice and Treatments:* The ApoE^−/−^ mice were raised at the First Affiliated Hospital of Anhui Medical University's Experimental Animal Center.

Mice were first adapted to high‐fat feeding for 1 week before surgery, narrowing their LCA.^30^ An adaptation period of 3 weeks was then used to establish a model of vulnerable plaque in mice fed a high‐fat diet. PBS, Fe_3_O_4_@PLGA, and PM/Fe_3_O_4_@PLGA were injected intravenously into mice 4 weeks after carotid artery constriction. LCA and RCA atherosclerotic lesions were detected via MRI after 24‐h.

*Recognition of vulnerable plaques* in vivo: Using a tail vein injection, mice bearing atherosclerosis‐vulnerable plaques were administered PBS, Fe3O4 nanoparticles, or PM/Fe3O4 nanoparticles (0.72 mg/kg Fe). In order to illustrate the cross sections of the LCA and RCA, MRI using a T2‐weighted sequence was performed using a BioSpec94/20 MRI scanner (echo time (TE):30 ms; repetition time (TR):3000 ms; Field of View (FOV) 25 mm × 25 mm; resolution 110 μm × 110 μm; slice Thickness 0.8 mm).

*H&E and Masson staining:* Under anesthesia, the mice LCA and RCA were carefully dissected, washed and fixed after sacrifice. For quantification of atherosclerotic lesions, the LCA and RCA were stained with ORO. For assessment of atherosclerotic plaque stability, Masson's trichrome was used on the LCA and RCA. The images were captured using OLYMPUS imaging software.

*Evaluation of inflammatory factors:* For assessing the inflammatory factors after MRI, mice were euthanized. Mouse serum was collected and inflammatory factors were detected using the ELISA kits.

*Statistical analysis:* The data are presented as the mean ± SEM. Statistical analysis was carried out using GraphPad Prism. One‐way analysis of variance (ANOVA) was utilized for data analysis. The significance level was defined as *p < 0.001, **p < 0.01 and ***p < 0.001.

## AUTHOR CONTRIBUTIONS

Conceptualization: Jun Xie and Yuyu Li; Methodology: Yuyu Li, Yujie Wang and Zequn Xia; Investigation: Yuyu Li, Daozheng Ke, Bing Song, Dan Mu and Ronghui Yu; Writing‐Original Draft: Yuyu Li; Funding Acquisition: Jun Xie; Resources: Dan Mu, Ronghui Yu and Jun Xie; Supervision: Yuyu Li and Jun Xie.

## CONFLICT OF INTEREST STATEMENT

The authors declare that they have no competing interests.

## ETHICS STATEMENT

All procedures with animals were approved by the Institution Ethics Committee of Anhui Medical University (Anhui, China) (No. LLSC20230846) and performed in accordance with the guidelines from Directive 2010/63/EU of the European Parliament.

## Supporting information

Supporting Information S1
