# Supplementary material for: Noninvasive platelet membrane‐coated Fe3O4 nanoparticles identify vulnerable atherosclerotic plaques
Source: Smart Med. 2024 Jun 4;3(2):e20240006. doi: 10.1002/SMMD.20240006 (PMC11235982; doi:10.1002/SMMD.20240006)
Supplement: Supplementary file 1 — Supporting Information S1 [file SMMD-3-e20240006-s001.docx]

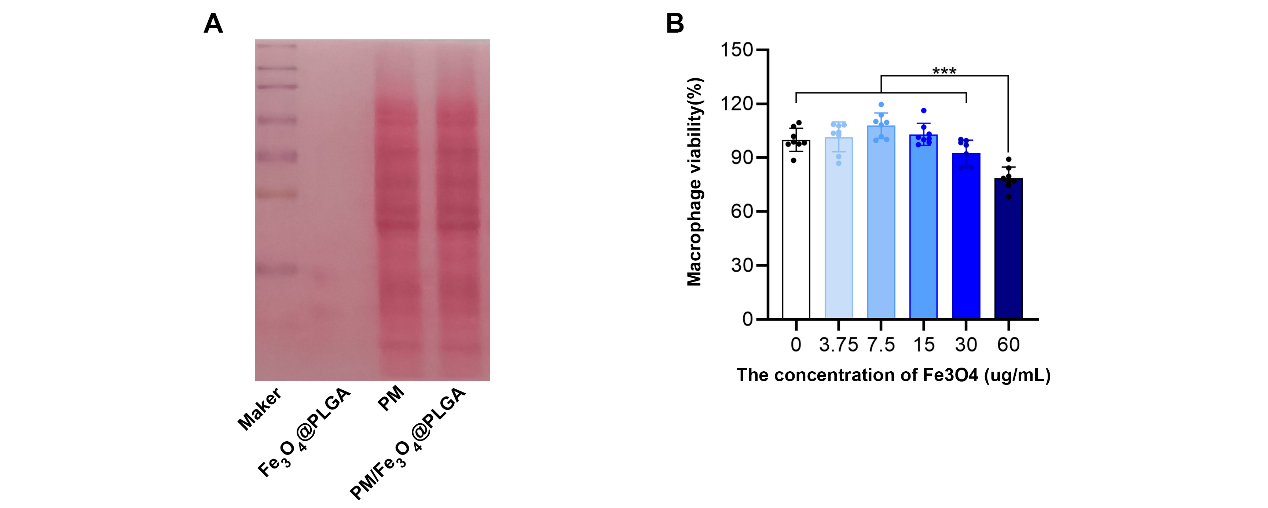


**Figure S1.** (A) Protein composition of Fe_3_O_4_@PLGA, platelet membrane and PM/Fe_3_O_4_@PLGA were shown by Ponceau dyeing. (B) Cell viability of macrophages after incubation with various doses of Fe_3_O_4_ nanoparticles for 24 h measured by CCK-8 (mean ± SD，n = 6).


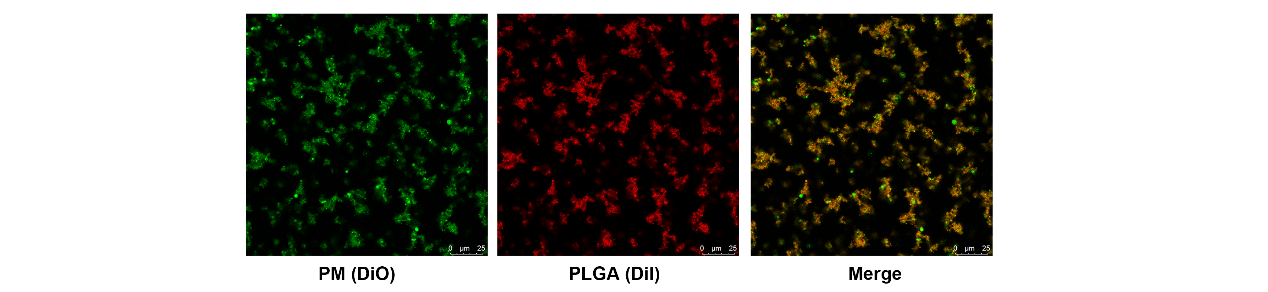


**Figure S2.** CLSM images of PM coated PLGA nanoparticles. The PM was labelled with DiO and the PLGA NPs were labelled with DiI (scale bar = 25 μm).


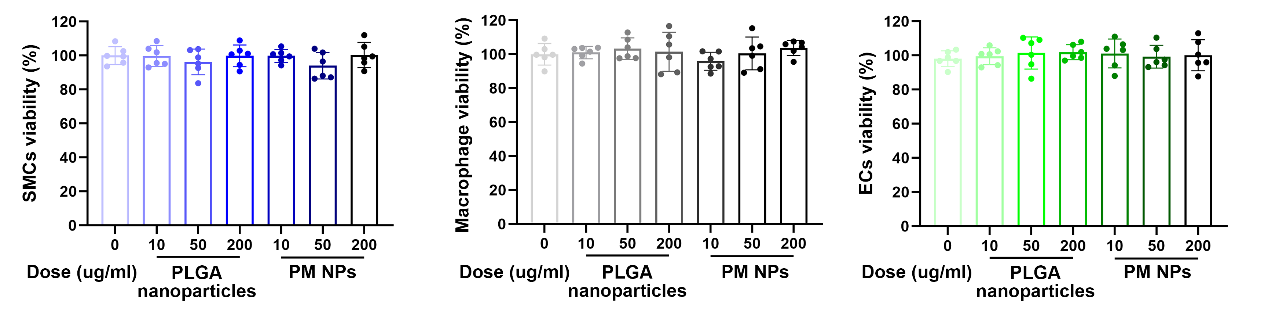


**Figure S3.** Cell viability of SMCs, macrophages and ECs after incubation with various doses of PLGA nanoparticles (PLGA NPs) and PM nanoparticles (PM NPs) for 24 h measured by CCK-8 (mean ± SD，n = 6).


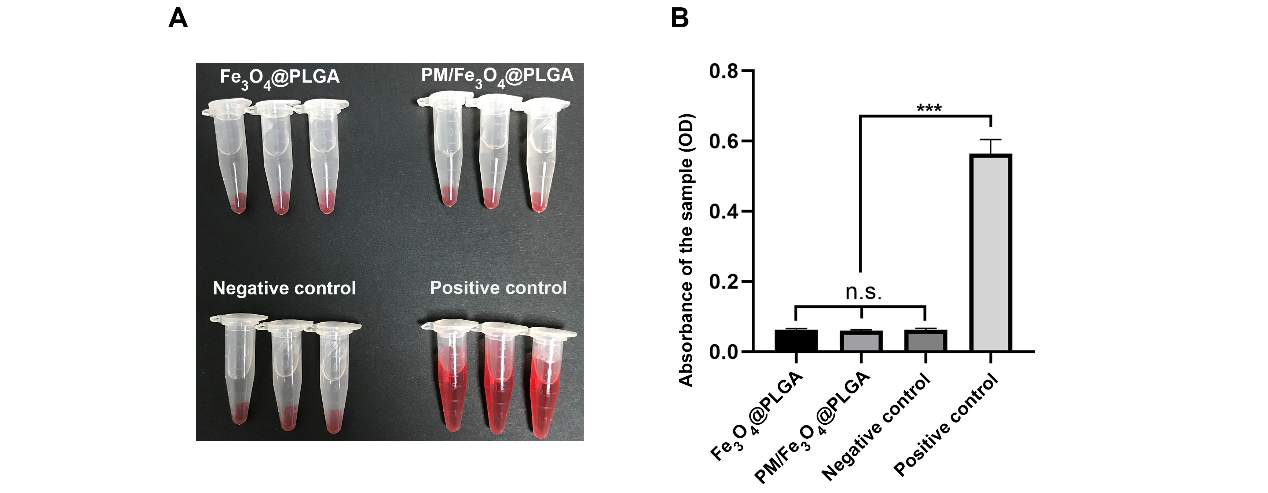


**Figure S4.** (A) Centrifuged red blood cells after incubation with Fe_3_O_4_@PLGA and PM/Fe_3_O_4_@PLGA. (B) The absorbance of the blood sample incubated with Fe_3_O_4_@PLGA and PM/Fe_3_O_4_@PLGA measured at 540 nm (n = 3, mean ± SD). ****p* < 0.001, n.s. = not significant, one-way ANOVA, Tukey’s multiple comparison test.


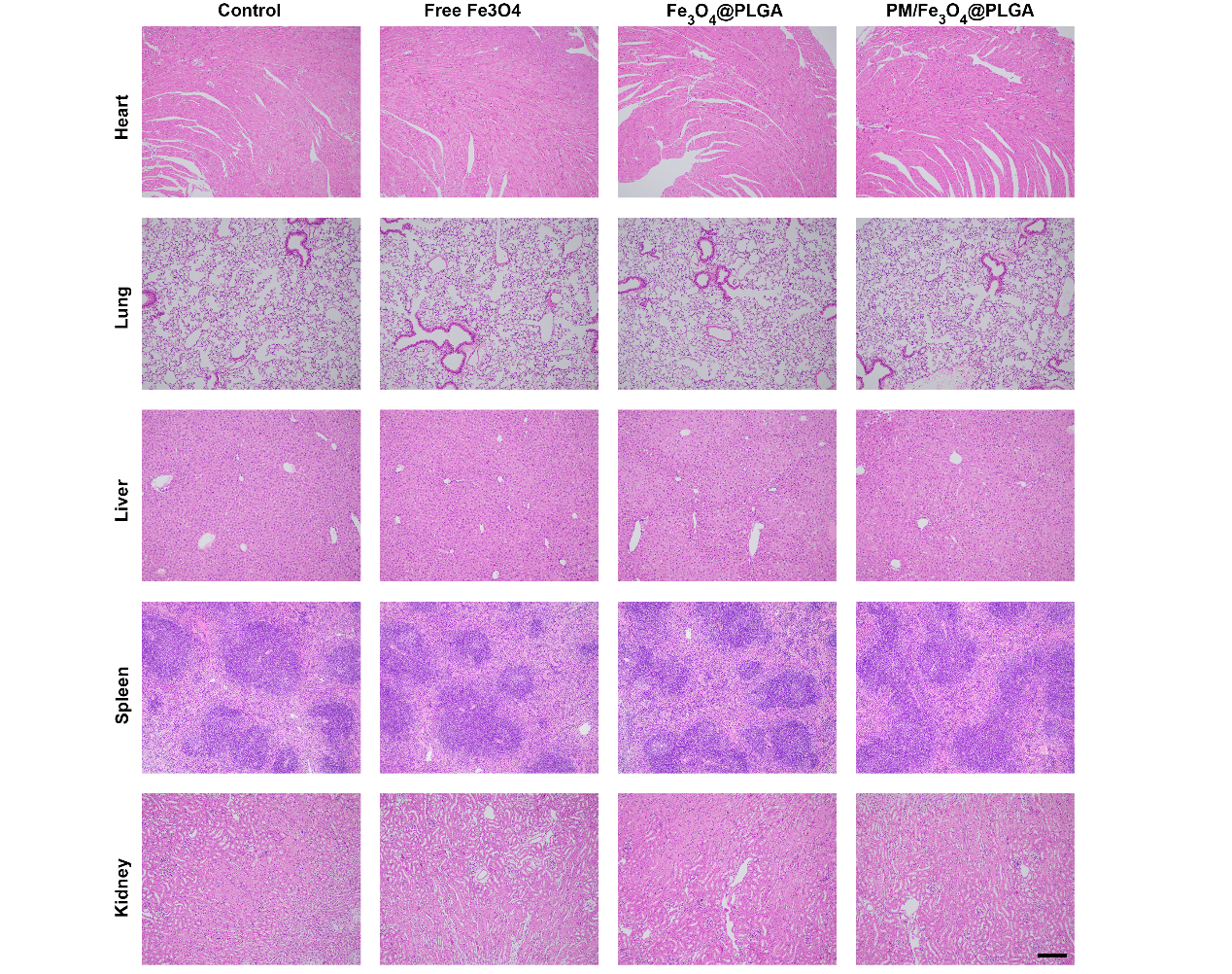


**Figure S5.** H&E staining of the main organs collected from the mice treated with free Fe_3_O_4_, Fe_3_O_4_@PLGA and PM/Fe_3_O_4_@PLGA (scale bar = 100 μm).


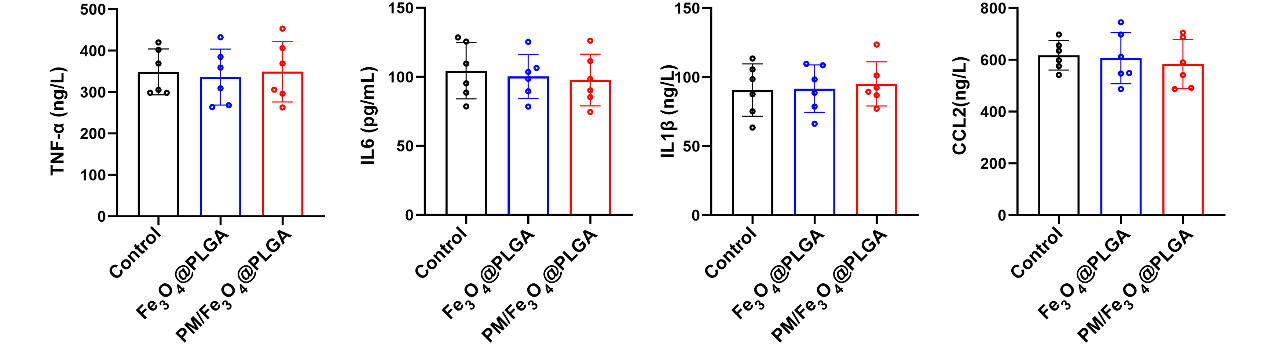


**Figure S6.** Mouse serum cytokines relevant to inflammation. (mean ± SD, n = 5 independent experiments). one-way ANOVA, Tukey’s multiple comparison test.
